# Phylogenetic Relationship of Plant *MLO* Genes and Transcriptional Response of *MLO* Genes to *Ralstonia solanacearum* in Tomato

**DOI:** 10.3390/genes11050487

**Published:** 2020-04-29

**Authors:** Jianlei Shi, Hongjian Wan, Wenshan Zai, Zili Xiong, Weiren Wu

**Affiliations:** 1 Fujian Provincial Key Laboratory of Crop Breeding by Design, College of Agriculture, Fujian Agriculture and Forestry University, Fuzhou 350002, China; sjlhebau@163.com; 2 Wenzhou Vocational College of Science and Technology, Wenzhou 325006, China; wenshanzai@sohu.com (W.Z.); xiongzili72@126.com (Z.X.); 3 Institute of Vegetables, Zhejiang Academy of Agricultural Sciences, Hangzhou 310021, China; wanhongjian@sina.com

**Keywords:** mildew resistance locus O (*MLO*), bioinformatics, gene duplication, phylogenetic relationship, homologous genes, *Ralstonia solanacearum*, gene expression

## Abstract

As a broad-spectrum disease resistance factor, *MLO* is involved in a variety of biotic and abiotic stress responses in plants. To figure out the structural features, phylogenetic relationships, and expression patterns of *MLO* genes, we investigated the genome and transcriptome sequencing data of 28 plant species using bioinformatics tools. A total of 197 *MLO* genes were identified. They possessed 5–7 transmembrane domains, but only partially contained a calmodulin-binding domain. A total of 359 polymorphic sites and 142 haplotypes were found in 143 sequences, indicating the rich nucleotide diversity of *MLO* genes. The *MLO* genes were unevenly distributed on chromosomes or scaffolds and were mainly located at the ends, forming clusters (24.1% genes), tandem duplicates (5.7%), and segment duplicates (36.2%). The *MLO* genes could be classified into three groups by phylogenetic analysis. The angiosperm genes were mainly in subgroup IA, *Selaginella moellendorffii* genes were in subgroup IA and IIIB, *Physcomitrella patens* genes were in subgroup IB and IIIA, and almost all algae genes were in group II. About half of the *MLO* genes had homologs within and across species. The Ka/Ks values were all less than 1, varying 0.01–0.78, suggesting that purifying selection had occurred in *MLO* gene evolution. In tomato, RNA-seq data indicated that *SlMLO* genes were highly expressed in roots, followed by flowers, buds, and leaves, and also regulated by different biotic stresses. qRT–PCR analysis revealed that *SlMLO* genes could respond to tomato bacterial wilt, with *SlMLO1*, *SlMLO2*, *SlMLO4,* and *SlMLO6* probably involved in the susceptibility response, whereas *SlMLO14* and *SlMLO16* being the opposite. These results lay a foundation for the isolation and application of related genes in plant disease resistance breeding.

## 1. Introduction

Various biotic and abiotic stresses are important factors that restrict plant growth and development. Meanwhile, plants have evolved effective defense mechanisms, in which resistance genes (*R* genes) play an important role in recognizing and resisting the invasion of pathogens. *MLO* (mildew resistance locus O) is the first powdery mildew resistance gene discovered in barley (*Hordeum vulgare* L.). Its recessive mutation leads to broad-spectrum, high-efficiency, and lasting resistance to different strains of powdery mildew. In addition to powdery mildew, *MLO* also participates in a variety of biotic and abiotic stress responses [1,2,3,4,5,6], revealing its great potential and broad prospects in plant resistance research.

Studies show that the *MLO* gene is located on the long arm of chromosome 4 in barley, with seven transmembrane helical domains (TMs) and one carboxyl terminal long tail [7]. Its N and C terminals are located extracellularly and intracellularly, respectively, and there is a calmodulin-binding domain (CaMBD) 10–15 amino acids residues away from TM7 [8]. In addition to barley, *MLO* homologs have also been identified in rice (*Oryza sativa* L.) [9], *Arabidopsis thaliana* [10], tomato (*Solanum lycopersicum* L.) [11], grape (*Vitis vinifera* L.) [12], cucumber (*Cucumis sativus* L.) [13], apple (*Malus domestica* Mill.) [14], pea (*Pisum sativum* L.) [15], cotton (*Gossypium hirsutum* L.) [16], poplar (*Populus trichocarpa* Torr. & Gray) [17], lentil (*Lens culinaris* Medic.) [18], pumpkin (*Cucurbita maxima* Duch.) [19], pepper (*Capsicum annuum* L.), and other monocots and dicots [20]. They are different from most of the *R* genes cloned previously.

*MLO* genes represent a new mechanism of broad-spectrum resistance caused by a host gene mutation. Although many plant *MLO* genes have been identified, few studies have been reported about the phylogenetic evolution of *MLO* genes and the *MLO* gene expression in response to *Ralstonia solanacearum*. In this study, we investigate *MLO* genes in different plant species, analyze their phylogenetic relationship, and analyze the interaction between *MLO* genes and *R. solanacearum* in tomato, aiming to provide basic data for further study of *MLO* gene function and underlying mechanisms and facilitate molecular breeding of disease resistance related to *MLO* genes.

## 2. Materials and Methods

### 2.1. Plant Species

Twenty-eight plant species with available genome sequence data, including nine angiosperm species (five dicots, three monocots, and one basalmost angiosperm), one gymnosperm species, one fern species, one bryophyte species, and 16 algae species, were investigated in this study (Table 1).

### 2.2. Identification of Plant MLO Genes

Two methods were used to retrieve the database: (1) The sequence in the conserved domain (PF03094) of *MLO* genes was downloaded from the Pfam database (pfam.xfam.org), and Blastp search (E-value ≤ 1e^−1^) was performed on Phytozome v12.1 (phytozome.jgi.doe.gov), NCBI (www.ncbi.nlm.nih.gov), and 1KP (https://db.cngb.org/onekp) databases. (2) The database was searched with the keyword “*MLO*”. The candidate genes were identified by Pfam based on the hidden Markov model (HMM).

Physicochemical parameters were calculated by the ProtParam program (web.expasy.org/protparam). TM, signal peptide, CaMBD, and subcellular localization were predicted using the TMHMM Server (www.cbs.dtu.dk/services/TMHMM), SignalP 4.1 Server (www.cbs.dtu.dk/services/SignalP), calmodulin-binding protein database (calcium.uhnres.utoronto.ca/ctdb/pub_pages/search/index.htm), and WoLF PSORT (www.genscript.com/wolf-psort.html), respectively.

### 2.3. Variation Analysis of Plant MLO Gene Sequences

The polymorphic information of gene sequences (variable site number, percentage of polymorphic sites, singleton variable sites, parsimony informative sites, and the total number of mutations) and haplotype diversity (haplotype, haplotype diversity, nucleotide diversity, and the average number of nucleotide differences) in different plants were analyzed by DnaSP 5.0 software.

### 2.4. Chromosome Localization of Plant MLO Genes

The chromosome map was made by the MapDraw v2.1 software based on *MLO* gene information. Subsequently, gene clusters and tandem duplication were analyzed. The criteria for determining gene clusters were (1) the distance between two adjacent *MLO* genes was less than 200 kb; and (2) the number of other genes between two adjacent *MLO* genes was no more than eight [21,22]. The criteria for tandem duplication were (1) the distance between adjacent *MLO* genes was less than 100 kb, and (2) the similarity between *MLO* genes was higher than 70% [23]. The gene synteny was examined by searching the Plant Genome Duplication Database (chibba.agtec.uga.edu/duplication/index/home), and the Circos diagram was drawn with TBtools [24].

### 2.5. Systematic Cluster Analysis of Plant MLO Genes

After extracting the amino acid sequences of *MLO* conserved domains and conducting multi-sequence alignment by the ClustalX 1.83 software, the phylogenetic tree was constructed using the maximum likelihood (ML) method implemented by MEGA 7.0 software with the JTT (Jones–Taylor–Thornton) model, a bootstrap value of 1000, and pairwise deletion. Each branch was displayed after removing the nodes with a bootstrap value of lower than 50%.The non-synonymous (Ka) and synonymous (Ks) base substitution rates and Ka/Ks values were calculated by PAL2NAL (www.bork.embl.de/pal2nal/index.cgi?example=Yes#RunP2N).

### 2.6. Promoter and miRNA Analysis of MLO Genes in Tomato

The 2000-bp upstream sequences of 17 *MLO* genes in tomato were downloaded from Solanaceae Genomics Network (solgenomics.net), and the cis-acting regulatory elements in these promoters were analyzed by the PlantCARE database (bioinformatics.psb.ugent.be/webtools/plantcare/html). miRNA targets were predicted based on mRNA sequences of *SlMLO* genes, using the miRBase 22.1 (www.mirbase.org) and psRNATarget (plantgrn.noble.org/psRNATarget) tools.

### 2.7. Digital Expression Analysis of MLO Genes in Tomato

Tomato RNA sequencing data were downloaded from the tomato functional genomics database (ted.bti.cornell.edu/cgi-bin/TFGD/digital/home.cgi). The gene expression heat-map was drawn, and the profile was analyzed by the MeV 4.9.0 software after removing low-quality data (*RPKM* < 1) and log2 standardization.

### 2.8. Expression Analysis of MLO Genes in Response to R. solanacearum in Tomato

The seeds of resistant and susceptible tomato lines, AH13112111 and G149351121, were sterilized, rinsed in sterile water, and sown in pots filled with a mixed matrix of peat, vermiculite, and perlite (2:1:1). When the fourth leaf appeared, the seedlings were subjected to *R. solanacearum* infection by root-soaking inoculation with a concentration of 10^8^ cfu/mL. Meanwhile, control seedlings were mock-inoculated with distilled water. They were then moved to a culture chamber with a 14 h/10 h diurnal cycle, 28/25 °C day/night temperature, and 80% humidity. After 48 hours, leaves were sampled, frozen in liquid nitrogen quickly, and kept at −80 °C for RNA isolation.

Total RNA was extracted from tomato leaves using a Trizol reagent (Sangon Biotech) according to the manufacturer’s instruction, and checked by RNA gel. The single-stranded cDNA was synthesized using a Maxima Reverse Transcriptase kit and used for quantitative real-time PCR (qRT-PCR). Three biological replicates were set, each with three technical replicates.

qRT-PCR was carried out in 96-well optical reaction plates using StepOne Plus Real Time PCR System (ABI, Foster, CA, USA). The *SlRPL2* (*Solyc10g006580.2.1*) gene was used as an internal control. The reaction mixture contained 2 μL cDNA, 0.4 μL PCR primer, 10 μL SYBR, and 7.2 μL ddH_2_O. The PCR ran for 45 cycles at 95 °C for 5 s and 60 °C for 30 s for anneal and extension. Gene-specific primers were designed by Primer Premier 5.0 (Table 2).

## 3. Results

### 3.1. Basic Characteristics of MLO Genes

A total of 197 *MLO* genes were identified from the 28 plant species (Table S1). The number of *MLO* genes in each species varied from one (*Picea sitchensis* and six algae species) to 26 (*P. trichocarpa*), with an average of seven. Among them, 30 *MLO* genes were in 16 algae species, 11 in *Physcomitrella patens*, 13 in *Selaginella moellendorffii*, 1 in *P. sitchensis*, 11 in *Amborella trichopoda*, 40 in three monocots, and 91 in five dicots.

The number of amino acids in MLO proteins ranged from 400 to 600. Some *MLO* genes had an N-terminal signal peptide and CaMBD. Most *MLO* genes were located in the cell membrane and contained 5–7 TMs.

### 3.2. Sequence Variation of MLO Genes

To evaluate the sequence variation of *MLO* genes, we examined 143 *MLO* genes from 14 representative species, which possessed integrated genome annotation information. In total, 359 (98.09%) variable sites were found in the CDS sequences of the 143 *MLO* genes, among which 19 (5.19%) were singleton variable sites, and 340 (92.90%) were parsimony informative sites. Meanwhile, 142 haplotypes were found in these *MLO* genes. The haplotype diversity, nucleotide diversity, and the average number of nucleotide differences among these *MLO* genes were 0.999 ± 0.0008, 0.42414, and 155.236, respectively.

Sequence variation of *MLO* genes also existed within species (Table 3; *P*. *sitchensis* was not listed because of incomplete data). The gene polymorphism site percentage ranged from 53.42 (*Volvox carteri*) to 85.22 (*P*. *trichocarpa*). The singleton variable sites and parsimony-informative sites ranged from 73 (*A. thaliana*) to 2591 (*Chlamydomonas reinhardtii*), and from 0 (two algae species) to 863 (*V. vinifera*), with an average of 442 and 615, respectively. The *MLO* gene polymorphism site percentage in dicots was higher than that in monocots, but the singleton variable sites were reverse. The algae species had lower polymorphism site percentage and contained only singleton variable sites. The number of mutations ranged from 1103 (*S*. *moellendorffii*) to 2591 (*C*. *reinhardtii*), with an average of 1771. The haplotype diversity in each species was about 1. The nucleotide diversity ranged from 0.41553 (*S. moellendorffii*) to 0.58474 (*C. reinhardtii*), with an average of 0.46123. The average number of nucleotide differences ranged from 364.000 (*S. moellendorffii*) to 2591.000 (*C. reinhardtii*), with an average of 737.821. The nucleotide diversity and the average number of nucleotide differences were larger in algae and had obvious differences from other species.

The results of the gene balance evolution test showed that the differences among species were not statistically significant (*p* > 0.10), and the *D* values were all negative, suggesting that plant *MLO* genes underwent mainly purifying selection (Table 4). The minimum recombination value of *MLO* gene loci in algae was 0, indicating that no recombination occurred in this region. However, the recombination values of other plant species were high, ranging from 88 (*S. moellendorffii*) to 156 (*A. trichopoda*), indicating that the recombination had a great influence on the nucleotide diversity of *MLO* genes.

The numbers of synonymous and non-synonymous substitution sites of *MLO* genes among 14 species were 87.51 and 278.49, respectively. Comparatively, the numbers of synonymous and non-synonymous substitution sites within species were much larger, varying from 203.06 (*S. moellendorffii*) to 1230.42 (*C. reinhardtii*) and from 672.94 (*S. moellendorffii*) to 3200.58 (*C. reinhardtii*), respectively (Table 4). The numbers of substitution sites in algae were more than those in other plant species. Fisher’s Exact Test indicated that the Ka/Ks values in different species were all less than 1 (Table 4). This was in line with the purifying selection and consistent with the nucleotide balance test.

### 3.3. Distribution of MLO Genes in Genomes

**Figure 1:**
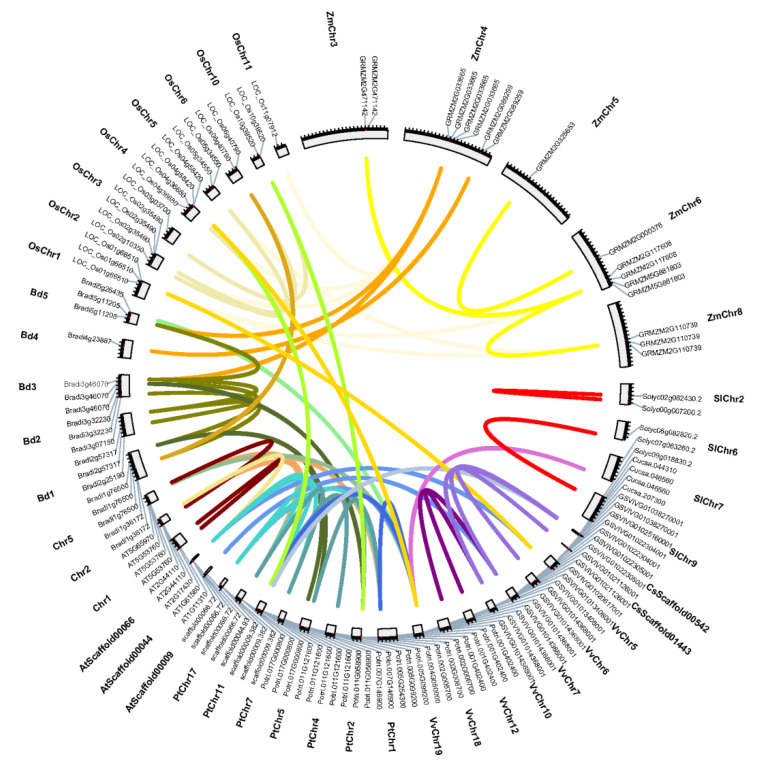
The synteny relationship of *MLO* genes among nine plant species. Bd, *B*. *distachyon*; Os, *O*. *sativa*; Zm, *Z*. *mays*; Sl, *S*. *lycopersicum*; Cs, *C*. *sativus*; Vv, *V*. *vinifera*; Pt, *P*. *trichocarpa*, and At, *A*. *trichopoda*. The gene pairs were linked by the lines between chromosomes.

Among the 197 *MLO* genes identified, excluding the 23 from algae that had no chromosomal location information, the remaining 174 *MLO* genes in 18 species showed a scattered distribution pattern across specific chromosomes or scaffolds and were mainly located at the ends (Figure S1). At most, there were five genes located on chromosomes 1 and 2 in *A. thaliana* and on scaffold00044 in *A. trichopoda*, respectively. A total of 19 gene clusters, containing 42 (24.1%) genes, were found in these species except for *Brachypodium distachyon*, rice, *S. moellendorffii,* and algae. There were six and five gene clusters in *P. trichocarpa* and grape, respectively, and five genes at most in one cluster on scaffold00044 in *A. trichopoda*. Conclusively, about 50% of *MLO* genes existed in clusters in the three plant species mentioned above. Furthermore, five pairs of tandem duplication genes were found in three species, with three pairs in *P. trichocarpa*, and one pair each in grape and *A. trichopoda*, which all appeared in gene clusters. Sixty-one pairs of segment duplicates involving 63 (36.2%) genes were found in nine species, indicating synteny relationships (Figure 1). Among them, 37 pairs were between species, with 11 between different monocots (six between rice and maize), 10 between different dicots, 8 between monocots and dicots, and 8 between *A. trichopoda* and dicots. In addition, 24 pairs were within species, with five in *B. distachyon*, four each in rice, maize, and *P. trichocarpa*, three in grape, and two each in tomato and *A. thaliana*. In short, there were more than 10 pairs of duplication genes in *P. trichocarpa*, grape, and three monocots, and the latter was up to 50–83.3%.

### 3.4. Phylogenetic Relationships of MLO Genes

**Figure 2:**
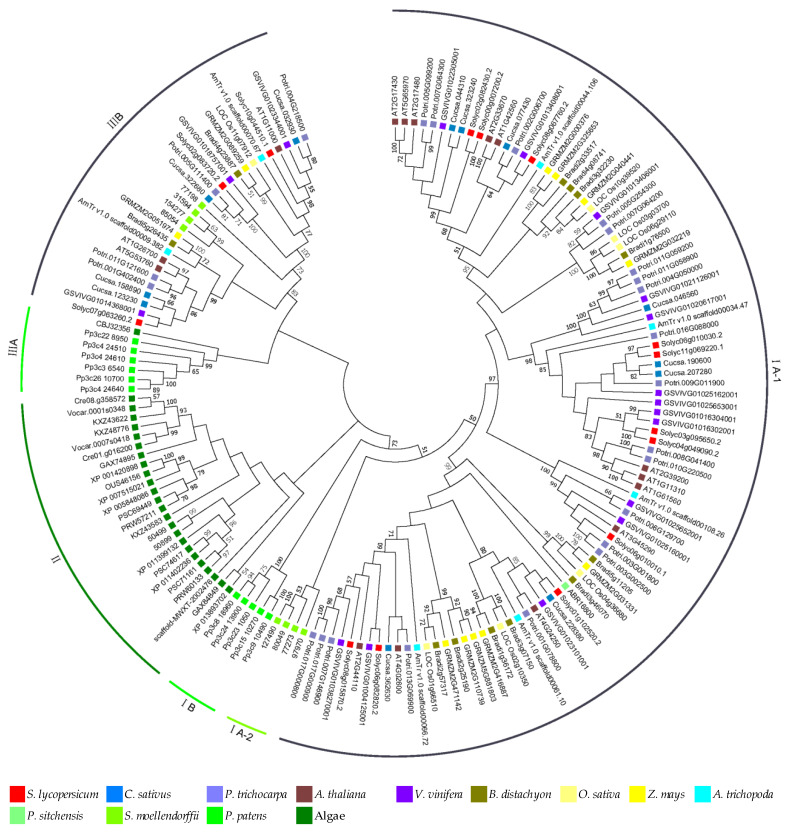
The phylogenetic tree of *MLO* genes in 28 plant species. The phylogenetic tree was constructed using the amino acid sequences of *MLO* conserved domains via maximum likelihood (ML) method. The selected 164 *MLO* genes were distributed on six clades.

In order to analyze the phylogenetic relationship of plant *MLO* genes, 164 *MLO* genes with an intact domain (excessively short sequences were excluded) from 28 species were used to construct a phylogenetic tree (Figure 2). These genes could be divided into three groups. Group I contained 105 (64.0%) genes, which could be further divided into three subgroups (I A-1, I A-2, and I B). Sixty-five (79.3%) dicot genes, 25 (83.3%) monocot genes, 5 (71.4%) *A. trichopoda* genes, and 1 *P. sitchensis* gene were in I A-1, 4 (50%) *S. moellendorffii* genes were in I A-2, and 5 (45.5%) *P. patens* genes were in I B, respectively. Group II contained 24 genes, all from algae. Group III contained 35 genes, which could be further divided into two subgroups (III A and III B). Six (54.5%) *P. patens* genes and 1 algae gene were in III A, and 17 (20.7%) dicot genes, 5 (16.7%) monocot genes, 2 (28.6%) *A. trichopoda* genes, and 4 (50%) *S. moellendorffii* genes were in III B, respectively. 

In addition, 25 pairs of orthologous genes were identified, of which 10, 8, 7, 6, 3, and 1 pairs were from *P. trichocarpa*, grape, *B. distachyon*, maize, algae, and *A. thaliana*, respectively, and four pairs each were from cucumber, rice, and *A. trichopoda*. Also, 28 paralogous genes were identified, of which eight and four pairs were in *P. trichocarpa* and *A. thaliana*, three pairs each were in tomato, maize and *P. patens*, two pairs each were in cucumber and *S. moellendorffii*, and one pair each was in grape, rice, and *B. distachyon*, respectively (Table S2). The results showed that 52.4% of the *MLO* genes had homologs, with *GSVIVG01014368001* having four, *Potri.001G402400*, *Potri.011G121600*, *Potri.011G058900* and *scaffold00009.382* each having three, and *Potri.005G254300*, *Potri.017G000800*, *AT5G53760*, *Cucsa.046560*, *Bradi2g57317*, *GRMZM2G089259*, *GRMZM2G110739*, *GRMZM5G881803,* and *LOC_Os01g66510* each having two, respectively. Among 53 pairs of homologous genes, 13 pairs were of segment duplicates, which were presumed to have a synteny relationship, and 4 pairs were of tandem duplicates. The Ka/Ks values of these homologous genes were all less than 1, ranging 0.01–0.78, suggesting that they underwent purifying selection.

### 3.5. Promoter Elements of SlMLO Genes

**Figure 3:**
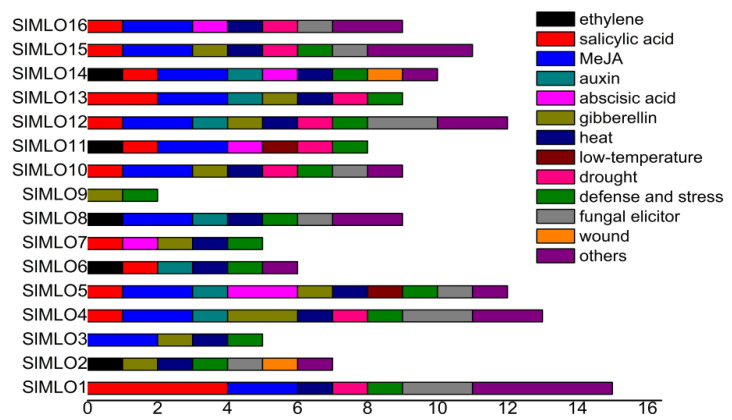
Cis-acting regulatory elements in the promoters of different *SlMLO* genes.

There were mainly three kinds of cis-acting regulatory elements related to hormone response, abiotic stress response, and resistance response in *SlMLO* gene promoters, including methyl jasmonate (MeJA), salicylic acid (SA), gibberellic acid (GA), indoleacetic acid (IAA), abscisic acid (ABA) and ethylene (ETH) response, heat and drought stress response, and elicitor recognition elements (Figure 3). *SlMLO1*, *SlMLO4*, *SlMLO5,* and *SlMLO12* contained more cis-acting elements that could respond to biotic and abiotic stresses. In general, the *SlMLO* genes contained 5–9 cis-acting elements, but *SlMLO9* only contained 2. Individual *SlMLO* genes contained regulatory elements such as cold and wounding responses and flavonoids biosynthesis.

### 3.6. miRNAs Targeting SlMLO Genes

Using the miRBase 22.1 and psRNATarget tools, 26 miRNAs were predicted to be able to target 12 *SlMLO* genes in tomato (Table 5). On average, one *SlMLO* was targeted by about two miRNAs, varying from one (*SlMLO3*, *SlMLO15,* and *SlMLO16*) to eight (*SlMLO12*). Most (18) of the miRNAs could only target one *SlMLO* gene, but there were seven (sly-miR156a, sly-miR156b, sly-miR156c, sly-miR172a, sly-miR172b, sly-miR9469-3p, and sly-miR6022) and one (sly-miR6027-5p) miRNAs that could target two and four *SlMLO* genes, respectively.

### 3.7. Digital Expression of SlMLO Genes

**Figure 4:**
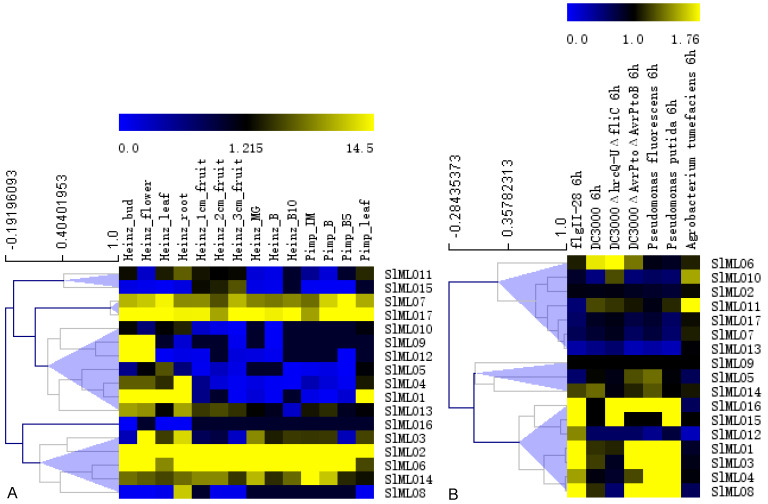
The expression of 17 *SlMLO* genes in different tissues (**A**) and under pathogen infection (**B**). (**A**) Expression in tomato cultivar Heinz and *Solanum pimpinellifolium*. MG, mature green; IM, immature green; B, breaker; B5, breaker + 5; B10, breaker + 10. (**B**) Expression in tomato leaves treated with different bacteria and PAMPs.

According to their expression patterns in different tissues, the 17 *SlMLO* genes could be divided into five groups (Figure 4A). *SlMlLO2*, *SlMLO6*, *SlMLO7*, *SlMLO14,* and *SlMLO17* were highly expressed in different tissues. *SlMLO1* was highly expressed in all tissues except fruits. *SlMLO3* was mainly expressed in flowers and roots. *SlMLO4* and *SlMLO8* were mainly expressed in roots. *SlMLO9* and *SlMLO12* were mainly expressed in buds and flowers. Overall, *SlMLO* genes were mainly expressed in roots, followed by flowers, buds, and leaves. 

In regard to their expression in response to pathogen infection, the 17 *SlMLO* genes could be divided into three groups (Figure 4B). *SlMLO1*, *SlMLO3*, *SlMLO4*, *SlMLO8,* and *SlMLO16* were highly expressed under different biotic stresses except for *Agrobacterium tumefaciens* infection. In contrast, *SlMLO10* and *SlMLO11* had a higher expression level under *A. tumefaciae* infection.

### 3.8. Transcriptional Response of SlMLO Genes to R. solanacearum

**Figure 5:**
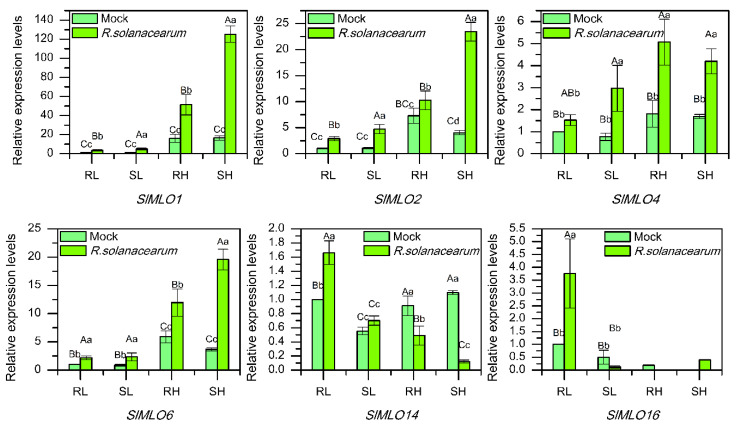
The relative expression levels of six *SlMLO* genes in resistant and susceptible tomato lines inoculated with *R. solanacearum*. R, resistant line; S, susceptible lines; L, leaf; H, whole seedling. Error bar indicates standard deviation. The uppercase and lowercase letters above the error bars indicate statistically significant differences (Tukey’s test, *p* < 0.01 and *p* < 0.05). The replication data of *SlMLO16* expression analysis in whole seedlings were missed.

Six representative *SlMLO* genes were selected for qRT-RCR analysis in light of their promoter elements and digital expression patterns. They all showed a significant response to *R. solanacearum* infection with different expression patterns (Figure 5). The expression of *SlMLO1*, *SlMLO2*, *SlMLO4,* and *SlMLO6* in leaf and whole seedling was all significantly upregulated after inoculation in both of the resistant and the susceptible tomato lines, but the response in the susceptible line was stronger than that in the resistant line in general. *SlMLO14* expression was upregulated in leaf but downregulated in whole seedling after inoculation in both lines. However, the degrees of expression change in the two lines were different. The upregulation in leaf was statistically significant only in the resistant line, while the downregulation in whole seedling was much more significant in the susceptible line than in the resistant line. *SlMLO16* displayed the opposite response to inoculation both between the two tissues (leaf vs. whole seedling) and between the two lines (resistant vs. susceptible). In leaf, its expression was upregulated in the resistant line but downregulated in the susceptible line; in whole seedling, the situation was just reversed. The above results implied that *SlMLO1*, *SlMLO2*, *SlMLO4,* and *SlMLO6* might be involved in the susceptibility response, while *SlMLO14* and *SlMLO16* might be involved in the resistance response.

## 4. Discussion

As a kind of negative regulatory factor, the recessively inherited mutation of *MLO* genes can enable plants to acquire broad-spectrum resistance to powdery mildew. In addition, it also participates in other biotic and abiotic stress responses, indicating that the identification and analysis of *MLO* genes are of great significance for plant resistance research. In recent years, systematic analysis and comparison of *MLO* genes have become possible with the completion of genome sequencing of a variety of plants.

### 4.1. MLO Genes had Specific Sequence Characteristics in Different Species

It has been shown that *MLO* genes originated at the early stage of land plant evolution [25]. They probably evolved in unicellular photosynthetic eukaryotes, and consolidated in land plants [26]. In this study, 197 *MLO* genes were identified from 28 species, including green algae and charophyte (Table S1), which support the above conclusion [27]. There was no deterministic relation between the number of *MLO* genes and the genome size in a species. For example, *P. sitchensis* had a larger genome, but only possessed one *MLO* gene, whereas *P*. *trichocarpa, V*. *vinifera,* and *A*. *thaliana* had smaller genomes but contained more *MLO* genes. In light of the number and size of *MLO* genes, it was speculated that extensive gene expansion, loss, and indels occurred in the process of plant evolution. Higher plant *MLO* genes contain 5–8 TMs, most of which were 7. However, it was slightly fewer in algae, with a large variation. In addition, *MLO* genes only partially had a CaMBD to bind with CaM to facilitate protein activity [8]. Most *MLO* genes were located in the cell membrane, and the function may be dependent on membrane signal transduction. The analyses of gene sequence polymorphism information and haplotype diversity showed that *MLO* genes had a rich genetic diversity (Table 3 and Table 4).

### 4.2. Duplication Was Widespread in Plant MLO Genes

In this study, *MLO* genes were found to be unevenly distributed on specific chromosomes or scaffolds, and mainly located at the ends (Figure S1), similar to other family genes. It is found that genes located at the ends may be easier to mutate in evolution [28,29]. Gene duplication is an important mechanism for plant gene family evolution. Tandem duplication genes are anchored in the same chromosome in clusters, and segment duplication genes are usually dispersed on different chromosomes [30]. It was found that 24.1% of *MLO* genes exist in clusters, and there were 5.7% and 36.2% tandem duplicates and segment duplicates, respectively (Figure 1 and Figure S1). All tandem duplication genes exist in clusters, suggesting that gene clusters and duplications are ubiquitous, and tandem duplication is an important way to form a gene cluster. This was obvious in *P. trichocarpa*, grape, and *A. trichopoda*. Segment duplication is more likely to appear in monocots. However, not all highly similar genes can form tandem duplicates. There may have been a gene insertion event during evolution. In addition, nearly 50% of duplication genes are in the opposite order on the chromosomes, suggesting that inversion may have occurred in these regions during plant evolution.

### 4.3. Numerous Homologs Were Ascertained in the Phylogenetic Relationships

This study showed that the identified *MLO* genes could be divided into three groups (I, II and III; Figure 2). Genes from angiosperms were mainly in subgroup I A, genes from *S. moellendorffii* were in subgroup I A and III B, genes from *P. patens* were in subgroup I B and III A, and almost all algae genes were in group II. It was speculated that *MLO* genes might have originated from higher algae and experienced different evolutionary processes in different species. *AT1G11310*, *AT1G61560*, *AT2G39200*, *Solyc04g049090.2*, *GRMZM2G032219*, and *LOC_Os06g29110* are known in function, so other genes in the same groups can be regarded as candidate resistance genes [31,32]. They provide important genetic resources for follow-up resistance breeding. Given that the six genes above were in different subgroups, it was speculated that the specific anti-powdery mildew function appeared after the differentiation of monocots and dicots. It showed the gene expansion in different species and chromosomes based on mixed branches with different genes. New gene features may arise due to changes to which the chromosome is subjected during evolution, such as recombination, replication, transposition, translocation, and deletion [33].

This study revealed that 52.4% of *MLO* genes had 1–4 homologs according to the sequence similarity, gene duplication, and phylogenetic relationship (Figure 1 and Figure 2, Table S2), suggesting that the doubling events had occurred in different degrees during evolution, but this proportion was less than that previously reported [34]. Only four pairs of homologous genes resulted from tandem duplication, which may be that a large number of duplication genes had function differentiation in evolution. There were 10 pairs of orthologous genes and 8 pairs of paralogous genes in *P. trichocarpa*. More than 50% genes had orthologous genes in *B. distachyon*. More than 50% genes had paralogous genes in *P. trichocarpa*, *A. thaliana*, *S. moellendorffii,* and *P. patens*, respectively. These results indicate that the expansion of most genes is specie-specific. This phenomenon is common in other plant gene families [28,29]. Increasingly resistant gene copies means enhanced gene function, but *MLO* homologous genes might have been lost in evolution.

While synonymous mutations do not change amino acid sequences, non-synonymous mutations are often deleterious. Therefore, the base substitution rate is lower under purifying selection. In this study, it was found that the Ka/Ks values of plant *MLO* homologous genes were all less than 1 (ranging 0.01–0.78; Table S2), indicating that the *MLO* genes underwent purifying selection in evolution. This was consistent with the nucleotide balance test. Deshmukh et al [31] also achieved the same conclusion. As mutant genes are usually at a disadvantage in selection and thus eliminated in the population, the evolution power may be from genetic drift [35].

### 4.4. MLO Genes Could Respond to Various Biotic Stresses in Tomato

It was found in this study that *SlMLO1*, *SlMLO2*, *SlMLO6*, *SlMLO7,* and *SlMLO14* were highly expressed in different tissues in tomato (Figure 4A). Some genes had the tissue expression specificity. For example, *SlMLO3* is mainly expressed in flowers and roots, *SlMLO4* and *SlMLO8* are mainly expressed in roots, and *SlMLO9* and *SlMLO12* are mainly expressed in buds and flowers. *SlMLO1*, *SlMLO3*, *SlMLO4*, *SlMLO8,* and *SlMLO16* could strongly respond to diverse biotic stresses (Figure 4B). Also, miRNA could potentially target one or more *SlMLO* genes (Table 5).

It was first found in barley that *MLO* genes have powdery mildew resistance and the recessively inherited mutation has high efficiency and lasting resistance to almost all physiological races of powdery mildew [7]. Besides, *MLO* genes can also participate in the responses to other diseases caused by *Hyaloperonospora arabidopsidis*, *Xanthomonas campestris*, *Magnaporthe oryzae*, *Pseudomonas syringae*, *Fusarium oxysporum*, and *Colletotrichum higginsianum*, as well as to abiotic stresses such as drought, salt, high and low temperatures [1,2,3,4,5,6], revealing the great potential and broad prospect in plant disease resistance research. In this study, for the first time, *MLO* genes were found to be able to respond to tomato bacterial wilt with diverse and complex expressions (Figure 5). It was speculated that *SlMLO1*, *SlMLO2*, *SlMLO4,* and *SlMLO6* may be involved in the susceptibility response, but *SlMLO14* and *SlMLO16* were the opposite. However, the direct function still needs molecular verification, and the related regulatory mechanism also needs further analysis.

Although gene expression can induce plant resistance, high expression of a large number of *R* genes is often lethal to plant cells. In view of this, coordinating *R* gene expression by small RNA (miRNA and siRNA) regulation is an important protective mechanism to reduce plant burden [36]. The cis-acting regulatory elements in promoters are not completely consistent with the actual gene expression level. On the one hand, the gene expression is related to diverse factors, on the other hand, many cis-acting elements may have not been identified [37,38].

## 5. Conclusions

*MLO* genes from 28 plant species were comprehensively analyzed based on the whole genome data and the bioinformatics method. A total of 197 *MLO* genes were identified, which were unevenly distributed on specific chromosomes or scaffolds, forming 19 gene clusters and 66 pairs of duplicates. These *MLO* genes could be classified into three groups by phylogenetic analysis. There were 25 pairs of orthologous genes and 28 pairs of paralogous genes. In tomato, some *MLO* genes were highly expressed in different tissues and under biotic stresses. For tomato bacterial wilt, *SlMLO1*, *SlMLO2*, *SlMLO4,* and *SlMLO6* appeared to be involved in the susceptibility response, *SlMLO14* and *SlMLO16* being the opposite. In short, plant *MLO* genes tend to exist in clusters, having evolved into a large number of homologous genes, and some genes can reversely respond to different stresses.

## Figures and Tables

**Table 1:** Plant species investigated in this study.

Species	Genome Size/Mb *	Lineage
*Solanum lycopersicum* L.	792.04	Dicot
*Cucumis sativus* L.	323.99	
*Populus trichocarpa* Torr. & Gary	434.29	
*Arabidopsis thaliana* (L.) Heynh	119.67	
*Vitis vinifera* L.	427.19	
*Brachypodium distachyon* (L.) Beauv.	218.35	Monocot
*Oryza sativa* L.	383.72	
*Zea mays* L.	2171.65	
*Amborella trichopoda* Baill.	706.50	Basalmost angiosperm
*Picea sitchensis* Engelm	18225.20	Gymnosperm
*Selaginella moellendorffii* Hieron.	212.32	Fern
*Physcomitrella patens* (Hedw.) Mitt.	472.08	Bryophyte
*Volvox carteri* F.Stein	137.68	Algae
*Chlamydomonas reinhardtii* P.A. Dangeard	120.41	
*Chlorella variabilis*	46.16	
*Chlorella sorokiniana*	58.61	
*Gonium pectorale* O.F. Müller	148.81	
*Monoraphidium neglectum*	69.71	
*Auxenochlorella protothecoides* (Krüger) T. Kalina & M. Puncochárová	22.92	
*Micractinium conductrix*	61.02	
*Chlamydomonas eustigma*	66.63	
*Micromonas* sp. RCC299	21.11	
*Micromonas pusilla* CCMP1545	21.96	
*Ostreococcus tauri*	13.90	
*Ostreococcus lucimarinus*	13.20	
*Bathycoccus prasinos* W. Eikrem & J. Throndsen	15.07	
*Chara vulgaris* L.	/	
*Ectocarpus siliculosus* (Dillw.) Lyngb.	195.81	

* The data were retrieved from NCBI (www.ncbi.nlm.nih.gov). /, data not found.

**Table 2:** Primers used for qRT-PCR.

Gene	Forward Primer (5′–3′)	Reverse Primer (5′–3′)
*SlRPL2*	GTCATCCTTTCAGGTACAAGCA	CGTTACAAACAACAGCTCCTTC
*SlMLO1*	GCAAACAGCAGACCAACCA	TTTCATTAGCCCACCCTTCA
*SlMLO2*	CGCGTGCTTGAAGCTGAT	GACCAAAGGGAACAAATGCTA
*SlMLO4*	CAAGGTCCTCTGTGGGTTCA	GCACGGATTATCGGTGTAGTT
*SlMLO6*	TGAATGTTAGCGGGTGGC	AAGGCAAAATGAATGAGGTGA
*SlMLO14*	GTGGGGATTTGTGGTGGG	AAGTTCGTCTCGTGGTTTTAGC
*SlMLO16*	TGGCTTCATTACGGCACAT	CTCCAACTTAGTCCCAATCACC

**Table 3:** Intra-species polymorphism and haplotype diversity of *MLO* genes.

Species	S	% S	SP	PIP	Eta	h	Hd	Pi	K
*Solanum lycopersicum*	863	82.66	94	769	1818	14	1.000 ± 0.027	0.45364	473.604
*Cucumis sativus*	747	80.06	98	649	1513	12	1.000 ± 0.034	0.44781	417.803
*Populus trichocarpa*	882	85.22	84	798	1878	24	1.000 ± 0.012	0.42523	440.112
*Arabidopsis thaliana*	863	81.49	73	790	1799	15	1.000 ± 0.024	0.44542	471.705
*Vitis vinifera*	942	81.35	79	863	2013	17	1.000 ± 0.020	0.43146	499.625
*Brachypodium distachyon*	885	79.73	137	748	1794	12	1.000 ± 0.034	0.43561	483.530
*Oryza sativa*	859	77.18	241	618	1502	7	1.000 ± 0.076	0.45463	506.000
*Zea mays*	745	78.59	110	635	1479	11	1.000 ± 0.039	0.43750	414.745
*Amborella trichopoda*	1106	77.61	257	849	2018	7	1.000 ± 0.076	0.47549	677.571
*Selaginella moellendorffii*	631	72.03	114	517	1103	8	1.000 ± 0.063	0.41553	364.000
*Physcomitrella patens*	870	78.80	112	758	1762	11	1.000 ± 0.039	0.45469	501.982
*Volvox carteri*	1750	53.42	1750	0	1750	2	1.000 ± 0.500	0.53419	1750.000
*Chlamydomonas reinhardtii*	2591	58.47	2591	0	2591	2	1.000 ± 0.500	0.58474	2591.000

S, number of variable sites; SP, singleton variable sites; PIP, parsimony informative sites; Eta, total number of mutations; h, number of haplotypes; Hd, haplotype diversity; Pi, nucleotide diversity; K, average number of nucleotide differences.

**Table 4:** Neutral testing and base substitution of *MLO* genes in different species.

Species	D	D *	F *	Rm	SS	NSS	Ka/Ks
*Solanum lycopersicum*	−0.77822	0.26621	−0.02567	102	237.08	806.92	0.2144
*Cucumis sativus*	−0.78581	0.27567	−0.00579	90	213.24	719.76	0.1617
*Populus trichocarpa*	−0.50659	0.58373	0.27683	97	237.70	797.30	0.1830
*Arabidopsis thaliana*	−0.65747	0.43878	0.15009	118	239.48	819.52	0.1896
*Vitis vinifera*	−0.69747	0.44997	0.13406	122	265.47	892.53	0.1714
*Brachypodium distachyon*	−0.88048	0.08421	−0.19513	115	257.26	852.74	0.2636
*Oryza sativa*	−1.02985	−0.14537	−0.38216	106	256.12	856.88	0.2535
*Zea mays*	−0.86823	0.05308	−0.20977	100	215.59	732.41	0.2635
*Amborella trichopoda*	−1.04622	−0.06242	−0.31490	156	321.55	1103.45	0.1958
*Selaginella moellendorffii*	−0.79390	0.09768	−0.12459	88	203.06	672.94	0.1530
*Physcomitrella patens*	−0.80473	0.12445	−0.13098	98	259.36	844.64	0.2215
*Volvox carteri*	/	/	/	0	854.17	2421.83	0.4734
*Chlamydomonas reinhardtii*	/	/	/	0	1230.42	3200.58	0.9794

D, Tajima’s D; D *, Fu and Li’s D *; F *, Fu and Li’s F *; Rm, minimum number of recombination events. SS, synonymous sites; NSS, nonsynonymous sites. /, no data available based on DnaSP operation rule.

**Table 5:** Predicted miRNAs targeting *SlMLO* genes in tomato.

Gene Name	Gene ID	miRNA ID	Accession no.	Mature Sequence
*SlMLO2*	*Solyc08g015870*	sly-miR396a-5p	MIMAT0035455	UUCCACAGCUUUCUUGAACUG
		sly-miR396b	MIMAT0035481	UUCCACAGCUUUCUUGAACUU
		sly-miR6027-5p	MIMAT0032133	AUGGGUAGCACAAGGAUUAAUG
		sly-miR167a	MIMAT0007917	UGAAGCUGCCAGCAUGAUCUA
		sly-miR167b-5p	MIMAT0035457	UAAAGCUGCCAGCAUGAUCUGG
		sly-miR1917	MIMAT0007909	AUUAAUAAAGAGUGCUAAAGU
*SlMLO3*	*Solyc06g010030*	sly-miR6027-5p	MIMAT0032133	AUGGGUAGCACAAGGAUUAAUG
*SlMLO4*	*Solyc00g007200*	sly-miR156a	MIMAT0009138	UUGACAGAAGAUAGAGAGCAC
		sly-miR156b	MIMAT0009139	UUGACAGAAGAUAGAGAGCAC
		sly-miR156c	MIMAT0009140	UUGACAGAAGAUAGAGAGCAC
		sly-miR482c	MIMAT0023603	UCUUGCCAAUACCGCCCAUUCC
*SlMLO5*	*Solyc03g095650*	sly-miR6027-5p	MIMAT0032133	AUGGGUAGCACAAGGAUUAAUG
		sly-miR9469-3p	MIMAT0035436	AUUCGGUCUUCUUAUGUGGAC
*SlMLO7*	*Solyc09g018830*	sly-miR172a	MIMAT0009143	AGAAUCUUGAUGAUGCUGCAU
		sly-miR172b	MIMAT0009144	AGAAUCUUGAUGAUGCUGCAU
		sly-miR1918	MIMAT0007910	UGUUGGUGAGAGUUCGAUUCUC
*SlMLO8*	*Solyc11g069220*	sly-miR6027-5p	MIMAT0032133	AUGGGUAGCACAAGGAUUAAUG
		sly-miR9470-3p	MIMAT0035440	UUUGGCUCAUGGAUUUUAGC
		sly-miR9478-3p	MIMAT0035474	UUCGAUGACAUAUUUGAGCCU
*SlMLO10*	*Solyc02g083720*	sly-miR6022	MIMAT0023590	UGGAAGGGAGAAUAUCCAGGA
		sly-miR9474-5p	MIMAT0035463	UGUAGAAGUCAUGAAUAAAAUG
*SlMLO12*	*Solyc08g067760*	sly-miR482e-3p	MIMAT0032124	UCUUUCCUACUCCUCCCAUACC
		sly-miR482d-5p	MIMAT0035459	GGAGUGGGUGGGAUGGAAAAA
		sly-miR156a	MIMAT0009138	UUGACAGAAGAUAGAGAGCAC
		sly-miR156b	MIMAT0009139	UUGACAGAAGAUAGAGAGCAC
		sly-miR156c	MIMAT0009140	UUGACAGAAGAUAGAGAGCAC
		sly-miR172a	MIMAT0009143	AGAAUCUUGAUGAUGCUGCAU
		sly-miR172b	MIMAT0009144	AGAAUCUUGAUGAUGCUGCAU
		sly-miR6024	MIMAT0023594	UUUUAGCAAGAGUUGUUUUACC
*SlMLO13*	*Solyc10g044510*	sly-miR6027-3p	MIMAT0023611	UGAAUCCUUCGGCUAUCCAUAA
		sly-miR156e-5p	MIMAT0035453	UGAUAGAAGAGAGUGAGCAC
		sly-miR9472-3p	MIMAT0035450	UUCACAAUCUCUGCUGAAAAA
*SlMLO14*	*Solyc07g063260*	sly-miR9469-3p	MIMAT0035436	AUUCGGUCUUCUUAUGUGGAC
		sly-miR1916	MIMAT0007908	AUUUCACUUAGACACCUCAA
*SlMLO15*	*Solyc02g077570*	sly-miR6022	MIMAT0023590	UGGAAGGGAGAAUAUCCAGGA
*SlMLO16*	*Solyc06g010010*	sly-miR6025	MIMAT0042023	UACCAAUAAUUGAGAUAACAUC

## Supplementary Materials

The following are available online at https://www.mdpi.com/2073-4425/11/5/487/s1. Figure S1: Chromosomal localization, gene clusters, and gene duplication of MLO genes. Table S1: MLO genes in different plant species and their sequence characteristics. Table S2: 53 pairs of homologous genes and their base substitution rates.
