# Supplementary material for: Phylogenetic Relationship of Plant MLO Genes and Transcriptional Response of MLO Genes to Ralstonia solanacearum in Tomato
Source: Genes (Basel). 2020 Apr 29;11(5):487. doi: 10.3390/genes11050487 (PMC7291212; doi:10.3390/genes11050487)
Supplement: Supplementary file 1 [file genes-11-00487-s001.zip › Supplementary Table S1 and S2 - revision.docx]

**Table S1.** *MLO* genes in different species and their sequence characteristics.

| **Code** | **Species** | ***Number of MLO Genes*** | **Exons *** | **ORF/bp *** | **Amino Acids *** | **Molecular Weight/kD *** | **pI *** | **Instability Index *** | **Grand Average of Hydropathicity *** | **Transmembrane Domains *** |
| --- | --- | --- | --- | --- | --- | --- | --- | --- | --- | --- |
| 1 | *Solanum lycopersicum* | 17 | 14 | 1435 | 477 | 54.64 | 8.63 | 39.79 | 0.121 | 6 |
| 2 | *Cucumis sativus* | 14 | 14 | 1553 | 517 | 60.18 | 8.94 | 41.50 | 0.041 | 6 |
| 3 | *Populus trichocarpa* | 26 | 14 | 1559 | 519 | 59.60 | 9.10 | 39.45 | 0.069 | 7 |
| 4 | *Arabidopsis thaliana* | 15 | 14 | 1612 | 536 | 61.39 | 8.96 | 39.88 | 0.045 | 7 |
| 5 | *Vitis vinifera* | 19 | 14 | 1505 | 501 | 57.39 | 8.96 | 41.86 | 0.041 | 6 |
| 6 | *Brachypodium distachyon* | 12 | 14 | 1566 | 521 | 58.75 | 9.13 | 37.33 | 0.125 | 7 |
| 7 | *Oryza sativa* | 12 | 12 | 1288 | 428 | 48.30 | 8.92 | 39.02 | 0.058 | 5 |
| 8 | *Zea mays* | 16 | 11 | 1326 | 441 | 49.90 | 9.22 | 35.95 | 0.057 | 5 |
| 9 | *Amborella trichopoda* | 11 | 11 | 1290 | 429 | 48.97 | 8.73 | 42.79 | -0.019 | 5 |
| 10 | *Picea sitchensis* | 1 | / | 1452 | 483 | 54.81 | 8.92 | 31.58 | 0.116 | 7 |
| 11 | *Selaginella moellendorffii* | 13 | 12 | 1143 | 380 | 43.73 | 8.88 | 38.97 | 0.314 | 6 |
| 12 | *Physcomitrella patens* | 11 | 14 | 1682 | 560 | 63.56 | 9.08 | 41.22 | -0.007 | 6 |
| 13 | Algae | 30 | 8 | 3143 | 949 | 100.86 | 7.57 | 47.75 | 0.025 | 6 |

***** The mean values of *MLO* family members for each species.

**Table S2.** 53 pairs of homologous genes and their base substitution rates.

| **Code** | **Gene 1** | **Gene 2** | **Sequence Similarity/%** | **Ka** | **Ks** | **Ka/Ks** |
| --- | --- | --- | --- | --- | --- | --- |
| 1 | AT1G11310 | AT1G61560 | 75 | 0.15 | 0.94 | 0.16 |
| 2 | AT1G26700 | AT5G53760 | 80 | 0.07 | 1.01 | 0.07 |
| 3 | AT1G42560 | AT2G33670 | 86 | 0.12 | 0.81 | 0.15 |
| 4 | AT2G17430 | AT5G65970 | 74 | 0.20 | 1.00 | 0.20 |
| 5 | Potri.001G402400 | Potri.011G121600 | 84 | 0.08 | 0.29 | 0.28 |
| 6 | Potri.003G001800 | Potri.003G002500 | 82 | 0.05 | 0.08 | 0.66 |
| 7 | Potri.005G099200 | Potri.007G064300 | 87 | 0.07 | 0.24 | 0.28 |
| 8 | Potri.008G041400 | Potri.010G220500 | 86 | 0.08 | 0.24 | 0.35 |
| 9 | Potri.011G058900 | Potri.011G059200 | 94 | 0.02 | 0.04 | 0.59 |
| 10 |  | Potri.004G050000 | 89 | 0.06 | 0.24 | 0.25 |
| 11 | Potri.017G000800 | Potri.017G000900 | 99 | 0.01 | 0.03 | 0.17 |
| 12 |  | Potri.007G146900 | 88 | 0.06 | 0.27 | 0.22 |
| 13 | Solyc00g007200.2 | Solyc02g082430.2 | 85 | 0.08 | 0.68 | 0.12 |
| 14 | Solyc03g095650.2 | Solyc04g049090.2 | 67 | 0.21 | 1.52 | 0.14 |
| 15 | Solyc06g010030.2 | Solyc11g069220.1 | 69 | 0.12 | 1.13 | 0.10 |
| 16 | GRMZM2G000376 | GRMZM2G325653 | 92 | 0.02 | 0.19 | 0.11 |
| 17 | GRMZM5G881803 | GRMZM2G110739 | 72 | 0.12 | 0.40 | 0.30 |
| 18 |  | GRMZM2G471142 | 68 | 0.19 | 0.92 | 0.21 |
| 19 | LOC_Os03g03700 | LOC_Os06g29110 | 78 | 0.10 | 0.54 | 0.19 |
| 20 | Cucsa.190600 | Cucsa.207280 | 77 | 0.14 | 1.72 | 0.08 |
| 21 | GSVIVG01016302001 | GSVIVG01016304001 | 79 | 0.13 | 0.17 | 0.78 |
| 22 | 80049 | 127490 | 81 | 0.03 | 0.05 | 0.61 |
| 23 | Pp3c4_24640 | Pp3c26_10700 | 72 | 0.17 | 0.71 | 0.24 |
| 24 | Pp3c8_18960 | Pp3c24_13900 | 59 | 0.30 | 0.81 | 0.37 |
| 25 | Pp3c9_10490 | Pp3c15_10270 | 65 | 0.25 | 0.97 | 0.26 |
| 26 | Cucsa.123230 | Cucsa.158890 | 79 | 0.12 | 0.95 | 0.13 |
| 27 | 154277 | 31594 | 67 | 0.21 | 9.55 | 0.02 |
| 28 | Bradi2g25190 | Bradi2g57317 | 72 | 0.19 | 0.91 | 0.21 |
| 29 | Bradi1g36172 | GRMZM2G416887 | 70 | 0.23 | 0.63 | 0.36 |
| 30 | Bradi5g11205 | GRMZM2G031331 | 76 | 0.17 | 1.10 | 0.16 |
| 31 | Bradi5g26435 | GRMZM2G051974 | 79 | 0.11 | 0.75 | 0.15 |
| 32 | Bradi3g07150 | LOC_Os02g10350 | 79 | 0.13 | 0.50 | 0.26 |
| 33 | Cucsa.322680 | Potri.005G111400 | 64 | 0.26 | 1.27 | 0.20 |
| 34 | Cucsa.032930 | Potri.004G218500 | 72 | 0.16 | 1.68 | 0.10 |
| 35 | Cucsa.046560 | Potri.011G058900 | 65 | 0.31 | 3.65 | 0.08 |
| 36 |  | GSVIVG01021126001 | 59 | 0.28 | 2.36 | 0.12 |
| 37 | GSVIVG01025652001 | Potri.006G129700 | 63 | 0.24 | 1.11 | 0.21 |
| 38 | GSVIVG01038270001 | scaffold00066.72 | 62 | 0.28 | 2.32 | 0.12 |
| 39 | GSVIVG01014368001 | Potri.001G402400 | 75 | 0.11 | 0.69 | 0.16 |
| 40 |  | Potri.011G121600 | 62 | 0.16 | 0.74 | 0.22 |
| 41 |  | AT5G53760 | 67 | 0.20 | 1.56 | 0.13 |
| 42 |  | scaffold00009.382 | 65 | 0.20 | 1.89 | 0.11 |
| 43 | Potri.005G254300 | GSVIVG01013406001 | 66 | 0.20 | 1.35 | 0.15 |
| 44 |  | Bradi1g76500 | 56 | 0.00 | 0.00 | / |
| 45 | GRMZM2G089259 | Bradi4g23887 | 82 | 0.11 | 0.66 | 0.17 |
| 46 |  | LOC_Os11g07912 | 80 | 0.10 | 0.69 | 0.14 |
| 47 | LOC_Os01g66510 | Bradi2g57317 | 79 | 0.13 | 0.49 | 0.27 |
| 48 |  | GRMZM2G110739 | 61 | 0.22 | 0.90 | 0.24 |
| 49 | scaffold00009.382 | Potri.001G402400 | 70 | 0.23 | 1.46 | 0.16 |
| 50 |  | Potri.011G121600 | 75 | 0.26 | 1.52 | 0.17 |
| 51 | Vocar.0001s0348 | Cre08.g358572 | 65 | 0.93 | 46.18 | 0.02 |
| 52 | PRW60133 | PSC71161 | 56 | 0.44 | 59.46 | 0.01 |
| 53 | OUS46156 | XP_001420898 | 65 | 0.21 | 5.66 | 0.04 |
